# DNAM-1 Activating Receptor and Its Ligands: How Do Viruses Affect the NK Cell-Mediated Immune Surveillance during the Various Phases of Infection?

**DOI:** 10.3390/ijms20153715

**Published:** 2019-07-30

**Authors:** Loredana Cifaldi, Margherita Doria, Nicola Cotugno, Sonia Zicari, Caterina Cancrini, Paolo Palma, Paolo Rossi

**Affiliations:** 1 Academic Department of Pediatrics, Research Unit of Congenital and Perinatal Infection, Bambino Gesù Children’s Hospital, 00165 Rome, Italy; 2 Department of Systems Medicine, University of Rome Tor Vergata, 00133 Rome, Italy

**Keywords:** NK cells, immune surveillance, DNAM-1, activating ligands, PVR, Nectin-2, viruses, viral life cycle phases

## Abstract

Natural Killer (NK) cells play a critical role in host defense against viral infections. The mechanisms of recognition and killing of virus-infected cells mediated by NK cells are still only partially defined. Several viruses induce, on the surface of target cells, the expression of molecules that are specifically recognized by NK cell-activating receptors. The main NK cell-activating receptors involved in the recognition and killing of virus-infected cells are NKG2D and DNAM-1. In particular, ligands for DNAM-1 are nectin/nectin-like molecules involved also in mechanisms allowing viral infection. Viruses adopt several immune evasion strategies, including those affecting NK cell-mediated immune surveillance, causing persistent viral infection and the development of virus-associated diseases. The virus’s immune evasion efficacy depends on molecules differently expressed during the various phases of infection. In this review, we overview the molecular strategies adopted by viruses, specifically cytomegalovirus (CMV), human immunodeficiency virus (HIV-1), herpes virus (HSV), Epstein-Barr virus (EBV) and hepatitis C virus (HCV), aiming to evade NK cell-mediated surveillance, with a special focus on the modulation of DNAM-1 activating receptor and its ligands in various phases of the viral life cycle. The increasing understanding of mechanisms involved in the modulation of activating ligands, together with those mediating the viral immune evasion strategies, would provide critical tools leading to design novel NK cell-based immunotherapies aiming at viral infection control, thus improving cure strategies of virus-associated diseases.

## 1. Introduction

Natural Killer (NK) cells have a pivotal role in innate immune protection against tumor development and viral infections [1]. NK cells, without prior sensitization, are able to recognize and kill virus-infected and transformed cells. The function of NK cells depends on the balance of signals triggered by receptors binding to activating or inhibitory ligands [2,3,4]. Several molecular defenses triggered by viruses or tumor cells on the surface of target cells include both the upregulation of ligands for activating receptors, and the downregulation of major histocompatibility complex (MHC) class I molecules [5], which are ligands for inhibitory receptors, thus mediating proper recognition and killing operated by NK cells and, definitely, promoting correct immune control. Moreover, as recently reported, in the murine tumor context, soluble ligands for activating receptors such as platelet-derived growth factor (PDGF)-DD and MULT1, secreted by cancer cells, were able to enhance NK cell-mediated antitumor functions [6,7]. This evidence revealed a new activating role of some excreted ligands, bringing up-to-date previous reports concluding instead that, generally, soluble ligands desensitize tumor cells to immune effector cells [8]. On the other hand, viruses adopt immune evasion strategies shared by tumor cells, including those affecting the NK cell-mediated immune surveillance [9,10]. Of note, chronic viral infections that, depending on the virus, can persist for the lifetime, also often trigger tumor development [11]. The impaired NK cell activity in both tumor and viral infections can be caused by dual mechanisms, on the one hand affecting the expression of NK cell-activating receptors, and on the other hand reducing the expression of ligands for activating receptors, thus rendering target cells less susceptible to NK cell-mediated recognition and killing [12,13].

NK cell-activating receptors include: (i) NK group 2 member D (NKG2D), belonging to NKG2 family (that includes also inhibitory receptors such as NKG2A and NKG2B) [14]; (ii) coactivating/adhesion DNAX-activating molecule (DNAM-1) [15]; (iii) natural cytotoxic receptor (NCRs) family that includes NKp30, NKp44 and NKp46 [16]; (iv) the killer immunoglobulin (Ig)-like receptors (KIRs) that are either activating or inhibitory, depending on their intracytoplasmic tail (short or long, respectively) [17]; and (v) the family of death receptors (DRs), including stimulating fragment (FAS) receptor and tumor necrosis factor-related apoptosis-inducing ligand (TRAIL) [18]. Notwithstanding the presence of activating receptors, the expression on NK cells of immune checkpoint molecules, such as cytotoxic T-lymphocyte antigen 4 (CTLA-4), programmed cell death protein 1 (PD-1), lymphocyte activation gene 3 (LAG-3), T-cell immunoglobulin domain and Mucin domain 3 (Tim-3), T cell immunoreceptor with Ig and ITIM domain (TIGIT), and CD96 renders NK cells functionally “exhausted” [19].

In particular, the DNAM-1 (CD226) receptor is known to be essential for NK cell–dependent antitumor immunity [20], and its role in immune responses to viral infections is emerging [21,22,23,24]. The expression of DNAM-1^bright^ has been detected in CD34^+^ precursor cells generating NK cells in patients with different chronic pathogen infections, such as those caused by HIV, HCV, tuberculosis (TB), and with some inflammatory diseases, representing a bone marrow (BM)-cell source ready to be mobilized in response to appropriate stimuli [25]. In the murine model, DNAM-1 expression regulates the generation of memory NK cells in response to CMV infection [23]. Of note, murine DNAM-1^-^ NK cells, that represent approximately half of all NK cells in uninfected state [26], increase during CMV infection and, in an experiment of adoptive transfer by using DAP12-deficient mice, transiently upregulated DNAM-1 expression shortly after CMV infection, reverting to a lower level later [23]. This plasticity was not so markedly observed in DNAM-1^-^ and DNAM-1^+^ NK cells separately injected in an alymphoid uninfected murine model. In that context, DNAM-1^+^ NK cells lost DNAM-1 expression, while DNAM-1^-^ NK cells were unable to re-express it [27], thus suggesting a differential DNAM-1 expression on NK cells in response to viral infection [23] or in relation with other immune components [27]. Of interest, DNAM-1^-^ NK cells, characterized by a great expression of NK-cell-receptor-related genes and producing high level of MIP1 chemokines, developed from DNAM-1^+^ NK cells that differently produced a higher level of inflammatory cytokines with respect to their negative counterpart, thus revealing a functional program of NK cell maturation marked by DNAM-1 expression [27].

The ligands for DNAM-1 are poliovirus receptor (PVR, also known as CD155 or nectin-like molecule 5) and Nectin-2 (CD112, or herpes entry mediator B—HVEB) belonging to the nectins and nectin-like protein family [15]. PVR has the higher affinity for DNAM-1 of those [28]. Ligands for DNAM-1 are expressed at low levels in several normal cells, such as epithelial, endothelial, neuronal and fibroblastic cells, and are up-regulated in response to cellular stress [29] and in many solid tumor cell lines of epithelial and neuronal origin, such as carcinomas, melanoma and neuroblastoma [30,31,32,33,34]. Of note, the expression of ligands for DNAM-1, for their peculiar role in cell adhesion and polarization, is relevant on cells infected by many viruses [35].

Viruses are able to evade the NK cell-mediated immune surveillance by several mechanisms, including the perturbed expression of DNAM-1 and/or its ligands, occurring especially during the late phase of infection. Herein, we report a brief summary of NK cell-immune escape strategies adopted by viruses, such as CMV, HIV-1, HSV, EBV and HCV, focusing on viral mechanisms, reported so far, leading to the modulation of DNAM-1 and its ligands.

## 2. DNAM-1 Signaling Pathway

The engagement of DNAM-1 (CD226) with its ligands is crucial for the susceptibility of tumors and virus-infected cells to NK cell-mediated killing. Indeed, DNAM-1 stimulation generates an activating signal that has been validated in many contexts [36,37,38,39,40]. Impairment of DNAM-1 triggering leads to increased tumorigenesis, as demonstrated by using DNAM-1 blockade antibodies and in a mouse model deficient for DNAM-1 expression. Moreover, binding of DNAM-1 with its ligands is involved not only in immune recognition, but also in several cell functions, such as cell proliferation, differentiation, adhesion, movement, polarization, and virus entry [41].

**Figure 1:**
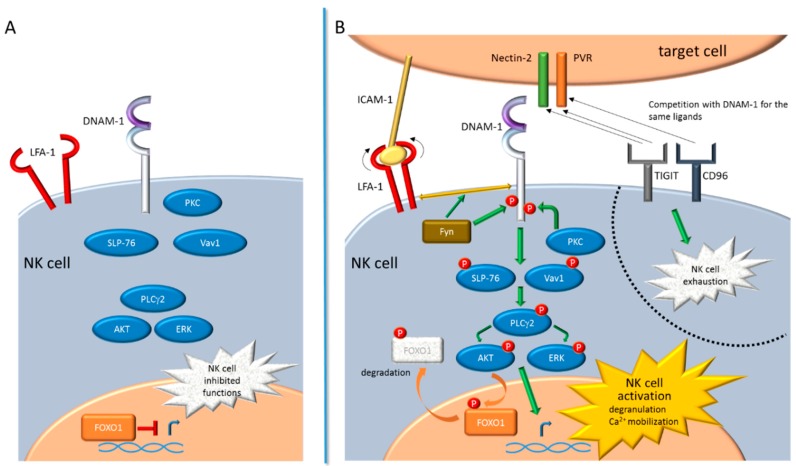
DNAM-1 signaling pathway leading to natural killer (NK) cell activation. (**A**) NK cell, not interacting with target cell, is not functional. FOXO1 negatively controls, at transcriptional level, many NK cell effector functions, including the cell homing and late-stage maturation. (**B**) When the immunological synapse is established between a NK cell and a target cell, several activating signal pathways trigger NK cell activation, including those mediated by DNAM-1. Upon the engagement of DNAM-1 with Nectin-2 or PVR activating ligands, expressed on the target cell, PKC phosphorylates the cytoplasmic domain of DNAM-1. At the same time ICAM-1, expressed on the target cell, binds LFA-1; thus promoting its conformational change; the recruitment of Fyn that phosphorylates the DNAM-1 cytoplasmic domain; and, as a consequence, the association of DNAM-1 with LFA-1. The DNAM-1 downstream signaling cascade leads to the phosphorylation of SLP-76 and Vav1, that induce the activation of PLCγ2 and, downstream, of ERK and AKT. This DNAM-1-dependent signaling cascade promotes the expression of genes involved in the degranulation and calcium (Ca^2+^) mobilization, leading to the NK cell activation. Activated AKT phosphorylates FOXO1, thus mediating the FOXO1 translocation from the nucleus to the cytoplasm, where it is degraded and inactivated. The expression of the immune checkpoint molecules TIGIT and CD96 renders NK cell functionally exhausted, since both receptors compete with DNAM-1 for the binding to the same activating ligands (indicated by black arrows).

DNAM-1 is a member of the immunoglobulin-like superfamily encoded by a gene located on chromosome 18 in humans. It is constitutively expressed at the cell surface of the majority of T cells, NK cells, and macrophages [24,42]. DNAM-1 is composed of a short leader sequence, an extracellular domain, a transmembrane domain, and a cytoplasmic domain. The cytoplasmic domain harbors a highly conserved sequence, with residues Y322 and S329 in human (equivalent to Y319 and S326 in murine ortholog) that, following ligand-engagement with the extracellular domain, can be phosphorylated [39]. The activating signal depends on the association of DNAM-1 with the lymphocyte function-associated antigen 1 (LFA-1) integrin, a heterodimeric molecule composed of an α1 chain (CD11a) and an β2 chain (CD18) [43]. Following the establishment of the activating synapse between a target cell and a NK cell, LFA-1 binds intercellular adhesion molecule 1 (ICAM-1) and undergoes a conformational change that sustains DNAM-1 activation. In more detail, in the murine model, upon the engagement of DNAM-1 with activating ligands, the S326 residue on the cytoplasmic domain is phosphorylated by protein-kinase C (PKC), allowing LFA-1 to associate with DNAM-1 through the Fyn tyrosine-protein kinase-mediated phosphorylation of the Y319 residue. Furthermore, the coordinated regulation of DNAM-1 with LFA-1 and their colocalization at the level of the activating synapse are essential events contributing to the functions of mature educated NK cells [44]. Afterwards, the DNAM-1 downstream signaling cascade leads to the phosphorylation of the lymphocyte cytosolic protein 2 (LCP2 or SLP-76) and Vav1, that induce the activation of phosphatidylinositol-4,5-bisphosphate phosphodiesterase gamma-2 (PLCγ2). Consequently, the extracellular signal-regulated kinase (ERK) and serine/threonine kinase (AKT) are activated [39], thus triggering the degranulation and calcium mobilization [24]. Moreover, activated AKT phosphorylates the FOXO1 transcription factor [45]. FOXO1 is known to be a negative regulator of NK cell homing, late-stage maturation, and effector functions [46]. When phosphorylated by AKT, nuclear FOXO1 translocates to the cytoplasm, where it is degraded and inactivated, thus supporting the NK cell activation (Figure 1).

DNAM-1-dependent NK cell activation is counterbalanced by two receptors belonging to the same immunoglobulin-like superfamily of DNAM-1: CD96 (also referred to TACTILE) [47] and TIGIT [48,49]. As opposed to DNAM-1, CD96 and TIGIT are inhibitory receptors that, by competing with DNAM-1 for binding to the same ligands, counteract NK cell activation [50]. In particular, CD96 binds PVR [51,52] while TIGIT binds both Nectin-2 and PVR [53,54] (Figure 1). Of note, several nectins are used as entry receptors by different human and animal viruses, thus the expression of both CD96 and TIGIT and their ability to compete with DNAM-1 for the engagement of such ligands are crucial in determining the fate of NK cells in mediating the anti-viral response [55].

## 3. Regulation and Function of DNAM-1 Ligands

Nectins, Ca^2+^-independent Ig-like molecules, represent a family composed by four members, nectins 1, 2, 3, and 4; the first three have two or three splice variants [41,56]. They are characterized by an extracellular region with three Ig-like loops, a single transmembrane region, and a cytoplasmic tail. The most distal extracellular Ig domain (D1) contains signature “lock” and “key” motif mediating molecule adhesion [57]. Nectins, as well as nectin-like proteins, function as cell adhesion molecules [58] involved in regulation of several cellular activities, including cell proliferation, survival, and movement, and of nervous and epithelial tissue polarization, development and organization [59,60]. 

Nectin-2 is a component of adherent junctions between epithelial cells [56,61], of Sertoli cell-spermatid junctions in the testis [62], and of the neuronal synapse [63], and also acts as an entry receptor for viruses [64,65]. Moreover, Nectin-2 is a regulator of endothelial cell proliferation and angiogenic function [66], and of cardiac structure and function [67].

PVR is a nectin-like molecule playing a critical role in cell adhesion and polarization [32]. PVR trans-interacts with Nectin-3 [68], and together with Nectin-2, trans-interacts with DNAM-1-associated LFA-1 [69]. Its overexpression promotes cell proliferation and migration [70].

The expression of Nectin-2 and PVR on tumors and virus-infected cells is induced through activation of several pathways, such as: (i) Damage DNA response (DDR) pathways, leading to the activation of ataxia telangiectasia mutated (ATM), and ATM and Rad3-related (ATR) protein kinases [71]; (ii) reactive oxygen species (ROS) production [72,73]; (iii) oncogenic Ras-mediating Raf-MEK-ERK-Ap1 signaling cascade [74]; (iv) Gli-mediated sonic hedgehog (Shh) signaling pathway [75]; (v) IFN-γ production [76].

## 4. Viral Modulation of DNAM-1 and Its Ligands

The immune evasion strategies adopted by several viruses include the downregulation of ligands for DNAM-1 that affect the NK cell-mediated recognition and killing of virus-infected cells, contributing to the development and the gravity of virus-related diseases.

Herein, we report the effect of five viruses, namely CMV, HIV-1, HSV, EBV, and HCV, that perturb the NK cell functions through many mechanisms, focusing on those regulating the expression of DNAM-1 and/or its cognate ligands, as summarized below.

### 4.1. Modulation by Cytomegalovirus (CMV)

Human CMV, a double-stranded DNA virus belonging to the *Herpesviridae* family, is frequently associated with salivary glands and establishes a life-long latency in healthy individuals. CMV infection causes severe disease and can be life-threating in immunocompromised hosts, such as newborn infants and subjects with primary immunodeficiency [77], acquired immunodeficiency syndrome (AIDS) patients [78], organ transplant recipients, and patients who have undergone hematopoietic stem cell transplantation (HSCT) [79]. As is the case with the majority of herpesviruses, after the infection human CMV remains latent throughout life and can be reactivated at any time. 

NK cells are recruited to the initial sites of a CMV infection to eliminate infected cells [80,81]. In general, people with defects in NK cell functions are extremely sensitive to herpesvirus infections, particularly to CMV. In addition, history of CMV infection has a deep effect on NK cells, with impact on memory and maturation phenotype within the NK cells which persists over time [82].

Mouse CMV is biologically similar to human CMV, thus it provides a useful tool to study CMV pathogenesis. In a murine model it has been demonstrated that both inflammatory monocytes and NK cells are essential in the early control of CMV infection, through a mechanism mediated by the binding of DNAM-1 with PVR expressed on virus-infected cells [83].

CMV contains genes with immunomodulatory functions able to induce several mechanisms leading to evasion of both the innate and adaptive immune responses. CMV efficiently downregulates MHC class I molecules, thus their failed engagement with inhibitory KIRs favors activating signals and consequently infected-cells become more susceptible to NK cell-mediated recognition and killing [84,85,86,87]. By contrast, several CMV proteins are able to block the functions mediated by NKG2D and DNAM-1 activating receptors, thus rendering viral-infected cells less susceptible to the elimination mediated by NK cells. Indeed, in CMV-infected cells the viral proteins UL16 [88,89,90], UL112, and UL142 [88,91,92,93] downregulate ligands for NKG2D, while the viral protein UL141 sequesters PVR in an intracellular compartment and blocks its expression at the cell membrane [94]. Moreover, UL141 downregulates Nectin-2 through the induction of proteasome-mediated degradation [95]. Specifically, the viral protein US2 supports UL141 in the retrotranslocation and degradation of Nectin-2 in the endoplasmic reticulum (ER) [96]. Similarly, in the murine model, the viral protein m20.1 affects the maturation of PVR in the ER, promoting its proteasome-mediated degradation, thus impairing dendritic and NK cell functions [83].

**Figure 2:**
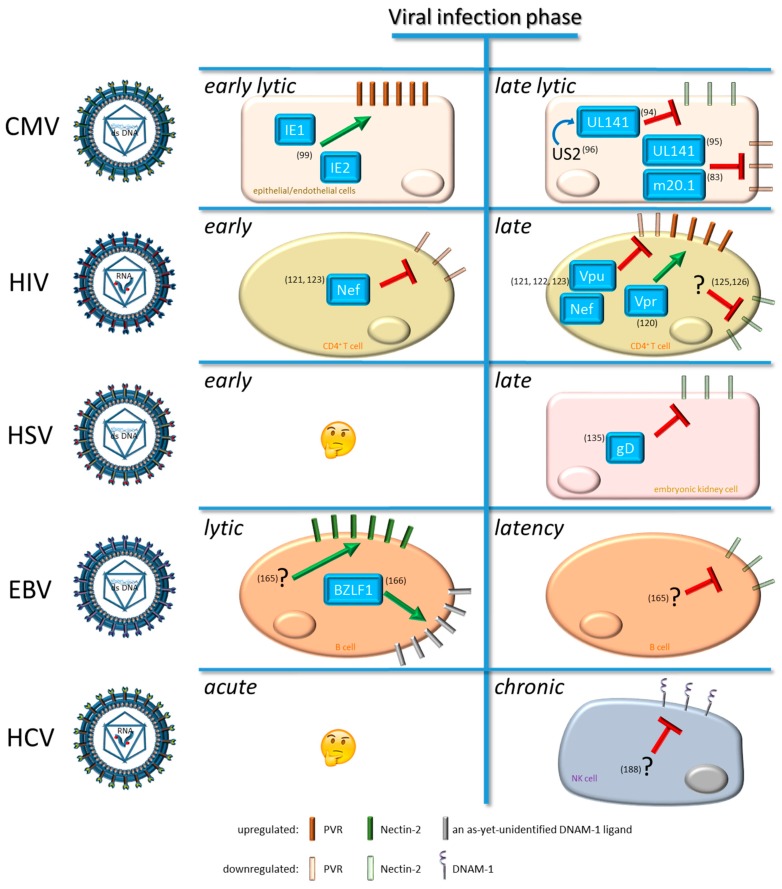
Viruses differently modulate the expression of DNAM-1 and their ligands depending on the infection phase. The viruses mentioned in this review (CMV, HIV-1, HSV, EBV, and HCV) differently express proteins associated with the various viral infection phases. Viral proteins (blue boxes) can upregulate (green arrow) or downregulate (red inhibition symbol) the expression of DNAM-1 and its ligands, such as PVR and Nectin-2, as reported in the legend. The US2 protein of CMV supports the immunomodulating function of UL141 during viral infection (blue arrow). References relative to each mechanism are reported in parenthesis. General missing information are indicated by emoticons, while missing information on viral molecules are indicated by question marks.

On the other hand, human CMV upregulates activating ligands such as MICA and ULBP-3 for NKG2D and PVR for DNAM-1. In particular, the major CMV immediate early (IE) proteins IE1 and IE2, known to be involved in the DDR pathway [97,98], stimulate the expression of both MICA and PVR [99]. PVR is upregulated by both IE proteins through a mechanism that does not require IE DNA binding activity and that deserves to be further investigated. This latter mechanism explains why CMV-infected cells in the early lytic phase could be eliminated by NK cells following viral expression of IE proteins. Notwithstanding, in the late lytic phase, CMV infected-cells express viral proteins involved in downregulation of ligands for DNAM-1 (Figure 2), thus promoting the NK cell immune-evasion that contributes to the viral latency. Then, NK cell-mediated control of CMV infection depends not only on the viral dissemination to a wide range of host tissues and cells, but also on the differential protein expression typical of each viral life cycle phases.

### 4.2. Modulation by Human Immunodeficiency Virus Type 1 (HIV-1)

HIV is a positive-sense, single-stranded RNA virus that belongs to the genus *Lentivirus* of the *Retroviridae* family, and can be classified in two types, HIV-1 and HIV-2. HIV-1 is a more virulent and infective strain than HIV-2, and is the cause of the global HIV infection, whilst HIV-2 is confined to West Africa. HIV infection is the main cause of AIDS [100]. A highly active combination of antiretroviral therapy (ART, cART, or HAART) that effectively controls HIV-1 viremia and viral transmission has dramatically reduced HIV-1-related morbidity and mortality [101]. Nevertheless, ART fails to eliminate HIV-1 due to the persistence of replication-competent proviruses in long-lived, latently infected T cells, also known as HIV-1 reservoirs [102], thus extensive efforts are devoted to find an effective cure for HIV-1 infection [103,104]. In the pediatric context, the model of early treatment showed a reduction of HIV viral reservoirs in isolated cases of post-treatment interruption of HIV viral control [105,106,107]. In line with this, we identified possible combined immune therapeutic interventions aiming at HIV-1 remission in perinatally HIV-1 infected children treated early after birth [108].

The principal target cells for HIV-1 are immune cells, such as helper CD4^+^ T cells, macrophages and dendritic cells [100]. HIV infected cells are recognized by innate immune cells, including NK cells and by CD8^+^ T cytotoxic cells. Natural HIV-1 infection is characterized by limited immune control of viral dissemination, severe depletion of CD4^+^ T cells, and a progressive impairment of both the acquired and innate arms of the immune system, causing increased susceptibility to opportunistic infections and contributing to the typical clinical scenario of AIDS [109].

The gravity of AIDS depends on how HIV is able to compromise the immune defense, including that mediated by NK cells. An increasing body of evidences shows that NK cell dysfunction is closely associated with HIV-1 disease progression, and that ART may not fully restore NK cell phenotype and activity [110,111]. In patients with chronic HIV-1 infection, an anergic CD56^-^ NK cell subset, characterized by high TIGIT expression, is expanded [55,112,113]. Moreover, several changes in the NK cell receptor repertoire have been described during HIV infection, including downregulation of activating receptors (e.g., NCRs and NKG2D) and increased expression of inhibitory receptors, leading to an impaired NK cell functionality [114,115,116]. As to DNAM-1 expression on NK cells of HIV-1-infected patients, conflicting data have been reported showing either no changes [117] or upmodulation of DNAM-1 levels [118,119]. Additionally, the impact of HIV-1 infection on the capacity of CD4^+^ T cells to express DNAM-1 ligands is controversial. Work performed in our laboratory demonstrated that, in the context of HIV-1-infected CD4^+^ T cells, PVR is exposed to dual post-transcriptional regulation exerted by the late Vpr viral protein, that increases PVR expression levels via activation of the DDR pathway [120], and by the early Nef and late Vpu viral proteins acting together at impeding PVR expression at the cell membrane [121]. As a net result, if compared to non-infected cells, HIV-1^+^ T cells express higher levels of PVR, hence are exposed to DNAM-1-mediated recognition and killing by NK cells [120,121]. These results were confirmed and extended by the work of other groups showing that the activity of Nef and Vpu, consisting of sequestering PVR in intracellular perinuclear compartments, was specifically evolved by the M group pandemic AIDS virus among different HIV-1 strains, and is conserved in patient-derived Nef and Vpu variants [122,123]. By contrast, another study showed that expression of PVR did not differ significantly on CD4^+^ T cells that were infected by wild type, Nef- and/or Vpu-deficient HIV-1, or those not infected [124]. Conceivably, the impact of HIV-1 proteins on PVR expression can be overlooked in experimental cell systems in which T cell activation, a prerequisite for permissive HIV-1 in vitro replication as well as a cause of PVR upregulation [72], is induced before [124] rather than after [121] HIV-1 infection. At any rate, in both studies the use of receptor-blocking antibodies in cytotoxicity assays showed that killing of HIV-1-infected T cells by NK cells is in part mediated by DNAM-1, although triggering of the NKG2D receptor is also required [121,124]. Controversial data were also reported regarding expression of the Nectin-2 ligand for DNAM-1 in HIV-1-infected CD4^+^ T cells. Indeed, decreased Nectin-2 expression was described on activated CD4^+^ T cells derived from HIV-1-infected patients, or in vitro infected cells, as compared to not infected control cells (Figure 2) [125,126]. Those results, however, are in sharp contrast with reported evidence clearly showing that Nectin-2 is not expressed on CD4^+^ T blasts [72], hence further investigations are needed.

### 4.3. Modulation by Human Herpes Simplex Virus (HSV)

Human HSV is a large, double-stranded linear DNA virus, consisting of a capsid containing the genome, surrounded by a lipid bilayer that forms the envelope. There are two types of herpes simplex virus, HSV-1 and HSV-2 that belong to the human *Herpesviridae* family. HSV-1 is transmitted by contact with infected persons, and causes cold sores or Herpes labialis, while HSV-2 is mainly sexually transmitted and causes Herpes vaginalis [127]. Both viruses are very contagious in humans, with 67% of the world population under the age of 50 being infected with HSV-1 and one out of eight individuals being infected with HSV-2 in the USA [128]. 

HSV-1 and-2 are neurotropic and neuroinvasive viruses, persisting in latently infected neurons and affecting the host immune surveillance. Upon reactivation from latency, the virus is transported by the nerve cells to the skin cells, where subsequent to cellular entry, it undergoes replication and shedding, and causes skin sores [129]. The virus’s cellular entry is regulated by four envelope glycoproteins involved in the fusion machinery: gD, gB and gH/gL [130]. The receptors for viral gD are Nectin-1 [131,132] and the Nectin-2 ligand of DNAM-1 [133]. Of note, as the main viral receptors on nerve and skin cells, Nectin-1 acts also for bovine herpes virus type 1, and simian B virus [134], while Nectin-2 acts also for porcine pseudorabies virus [135].

NK cell functions are crucial in controlling HSV-1 and -2 infection [136,137,138,139,140] through interaction with dendritic cells [141]. HIV/HSV-2 co-infected subjects have NK cells potentiated in terms of cell number as well as cytotoxicity [142]. Patients affected by atopic dermatitis are potentially susceptible to HSV infection, due to the impaired NK cell functions, as evaluated in a HSV-infected murine model [143]. Patients suffering recurrent HSV-1 reactivation have NK cells expressing significantly decreased levels of a panel of activating receptors, although DNAM-1 was not investigated [144]. Moreover, NK cells in response to recurrent HSV-2 reactivation, differently to CMV, hantavirus, chikungunya virus and HIV-1 infections, do not highly differentiate in peripheral blood [145]. HSV evades the immune-mediated control through many mechanisms, not only by interfering with MHC class I antigen presentation, but also by downregulating the expression of activating ligands for NKG2D [146] and DNAM-1 [135] on infected cells. Nectin-1 is downregulated within HSV infected cells, since newly synthesized late structural protein gD, accumulated at cell contact sites during the lather phase of HSV infection [147], competes for the canonical adhesive site [148,149,150], inducing ligand endocytosis, an event that correlates with endocytic HSV entry [151,152]. Nectin-2 is downregulated by HSV-2 gD, thus preventing DNAM-1-mediated killing of HSV^+^ cell targets by NK cells (Figure 2) [135]. The control of infection and viral entry can be mediated by using fusion proteins containing the entire ectodomain of herpes virus mediator entry (HVEM), Nectin-1, Nectin-2 and the Fc portion of human IgG, as evaluated in a murine model of ocular herpes infection [153]. Interestingly, for all these intrinsic proprieties linked to adhesion, entry and invasive mechanisms, HSV has been proposed for oncolytic purpose in NK cell-mediated immunotherapy [154,155].

### 4.4. Modulation by Epstein-Barr Virus (EBV)

EBV, a double-stranded linear DNA virus, is a member of the *gamma-herpes* virus family and affects more than 90–95% of the global population. EBV infection, known to be the first cause of mononucleosis (IM), also called “kissing disease,” represents one of the risk factors for the development of Multiple Sclerosis (MS) [156] and is associated with several malignant diseases such as Burkitt’s lymphoma [157], or nasopharyngeal carcinoma [158], and particularly in patients with primary immunodeficiencies [159]. EBV is transmitted via saliva exchange and firstly infects epithelial submucosal cells in which viral replication occurs, then infects naïve memory B cells that differentiate into activated B cells and plasma cells [160].

NK cells play a critical role in controlling EBV infection [161], mainly promoting the killing of infected-B cells during the viral lytic phase [162,163]. Acute IM is characterized by a fivefold expansion of an early-differentiated NKG2A^+^KIR^-^CD57^-^ NK cell subset, preferentially proliferating and functioning on exposure to EBV-infected B cells expressing lytic antigens [164]. The preferential NK cell-mediated killing of infected cells in lytic phase has been linked to EBV-induced upregulation of both ULBP-1 and Nectin-2 on infected-cells [165]. On the other hand, latently EBV-infected cells were shown to be negative for both ULBP-1 and Nectin-2 expression [165] (Figure 2), explaining the reasons for an efficient NK cell-mediated control in the early EBV infection phase and its failure in the latency phase.

Another study showed that the BZLF1 lytic protein exclusively induces ULBP-2 expression, yet triggers NK cell killing in a DNAM-1-dependent manner, suggesting the induction of an as-yet-unidentified DNAM-1 ligand (Figure 2) [166]. In these controversial studies, data on ligand regulation did not always match with the response of cognate activating receptors, therefore further investigations are warranted to better understand the DNAM-1-dependent NK cell functions in response to EBV infection.

The selective NK cell inability to kill EBV-infected B cells may also result from inhibitory signals, as demonstrated in the context of X-linked lymphoproliferative disease [167]. Of note, EBV early after infection of primary B cells, leading to the transformation into lymphoblastoid cell lines (LCLs), potently up-modulates the expression of DNAM-1 at both mRNA and protein levels [168]. DNAM-1, normally not expressed on B cells, is instead high on EBV-infected B cells in the latency III stage, but is low in those in latency 0 or I growth programs, suggesting that the role of DNAM-1 in cellular aggregation forming “clumps” can confer a survival advantage to EBV-infected cells [168].

All that evidence suggests that, in order to better understand the role of NK cells in controlling EBV infection and contrast the developments of EBV-associated malignancies, a deeper exploration of the EBV-mediated modulation of ligands for NK cell-activating receptors is warranted.

### 4.5. Modulation by Hepatitis C Virus (HCV)

HCV, one of the etiological agents of viral hepatitis, is a single-stranded RNA virus belonging to the genus *Hepacivirus* of the *Flaviviridae* family. HCV infection is usually asymptomatic, resulting in viral persistence with a dysregulation of the immune system, and in late diagnosis during the development of a serious chronic liver damage [169,170]. 

NK cells play an important role in controlling HCV infection and are resultantly functionally altered in chronic HCV patients [171,172,173,174,175]. Genetic studies revealed that patients able to spontaneously eliminate HCV are homozygotes for KIR2DL3 and its HLA-C1 ligand [176,177,178]. Moreover, the expression of both activating receptors NKp30 [179] and TRAIL [180] correlates with NK cell efficiency in protecting from HCV infection. HCV affects NK cell activity through many mechanisms [181,182], such as that mediated by the viral E2 protein that, by binding to NK cell-CD81 receptor, reduces the production of both IFN-γ and cytotoxic granules [183,184]. Moreover, HCV induces the down-regulation of ligands for NKG2D [185]. NK cells from HCV-patients, even if expressing high levels of NKp30 and NKp46, produce high levels of IL-10 [115]. This cytokine stimulates the secretion of transforming growth factor (TGF)-β and downregulates NKG2D on the NK cell surface [186], thus contrasting the immune-mediated viral clearance in the liver’s microenvironment. Moreover, humanized C/O^Tg^ mice, permissive for persistent HCV infection, have exhausted NK cells, caused by the upregulation in hepatocytes of ligands for NKG2A inhibitory receptor [187]. Chronically HCV-infected patients, showing a sustained virological response, had NK cells expressing lower levels of NKp30, ILT2/CD85j and DNAM-1 (Figure 2), and higher levels of NKG2A compared with healthy donors [188]. Of note, IFN-α-activated NK cells require DNAM-1 to successfully recognize and kill HCV-infected hepatoma cells, promoting the reduced viral replication in the infected cells [189].

While the involvement of cytokines in the HCV-mediated regulation of NK cells has been more extensively evaluated, the modulation of ligands for activating receptors and, in particular, for DNAM-1, in the liver diseases associated to HCV infection, is still needed. A further exploration should be of high interest, including in the context of the HCV NK cell-mediated control on the hepatocellular carcinoma onset [190].

## 5. Conclusions and Future Perspectives

The physiological expression of activating receptors represents a natural weapon allowing an effective and daily NK cell-mediated line of defense against viral infections [191]. The persistence of viral infections is associated very often with the impaired NK cell functions occurring in many disease conditions. Viruses adopt several molecular immune escape strategies, including those affecting the expression of activating receptors on NK cells and/or that of ligands for activating receptors on virus-infected cells.

DNAM-1, for its implication in cell proliferation, adhesion and polarization, is an activating receptor particularly important in the context of viral infection. Its ligands, belonging to the nectin family, are receptors for several viruses that mediate both the viral particle attack and entry, allowing the infection of cells of different origins. Interestingly for HSV, the expression of nectins on target cells on one side represents an advantage for the infected host in mediating the DNAM-1-dependent NK cell recognition and killing, but, on the other side, allows the entry of virus into nerve and skin cells. Moreover, adverse events occurring in several diseases, that compromise DNAM-1-dependent NK cell function and migration in the viral infection site, generally favor virus proliferation and dissemination. Thus, the NK cell-mediated anti-viral control depends on many mechanisms, including the virus’s cycle phase, the viral load, the NK cell status/responsiveness to viral infection and the NK cell recruitment to the site of infection [192].

One of the major limits of NK cells is their inability to radically eliminate virus infected cells due to the intrinsic ability of several viruses to cycle from the lytic to the latent phase. Indeed, in the latent phase, viral protein transduction is strongly reduced, thus rendering virus-infected cells less susceptible not only to NK cell-mediated attack, but also to other immune components [193]. Viruses such as CMV, HIV-1, HSV, and EBV, cycling from the early to the late phase, express proteins differently involved in the regulation of ligands for NK cell-activating receptors. During the early lytic phases, viruses induce the expression of ligands for NK cell-activating receptors, including those for DNAM-1, thus conferring the susceptibility of virus-infected cells to NK cell-mediated recognition and killing. By contrast, when a viral latency program is triggered, viral molecules associated with this late phase induce the downregulation of activating ligands, including those for DNAM-1, thus contributing to the viral immune evasion (Figure 2). The switch from upregulation to downregulation of DNAM-1 ligands upon progression from early to late phase of infection is particularly evident for CMV, EBV and HSV, which are viruses following finely regulated viral life cycle programs; but not HIV-1 for which it is challenging to distinguish an early and a late phase due to intrinsic viral replication features.

Among the various immune evasion strategies adopted by viruses, the shedding of ligands for DNAM-1, occurring in many tumor contexts [194], is still unexplored. Of note, differently to initial reports depicting negative immune effects associated with soluble ligands [8], the MULT1 ligand for NKG2D and PDGF-DD ligand for NKp44 are able to promote the NK cell activation [6,7]. Interestingly, CMV infection induces in glioblastoma cells the secretion of PDGF-DD—a growth factor promoting pericyte recruitment—angiogenesis, and tumor growth [195]. PDGF-DD, being a ligand for NKp44 [6], potentially sensitizes, in this viral context, tumor cells to NK cell recognition and killing. Then, since NK cell activating receptors can cooperate in mediating NK cell functions [196], viral mechanisms involved in the induction of soluble ligands for activating receptors, synergizing with DNAM-1, merit more extensive study.

Molecular strategies aimed to boost the NK cell-mediated anti-viral response are still under investigation [197]. Latency reversing agents (LRAs), known as drugs able to induce viral production and unmask latently-infected cells, represent a promising clinical approach to cure HIV-1 [198,199]. LRAs promote “shock-and-kill” immune-mediated mechanisms, including those dependent on NK cells [200,201,202]. Interestingly, in the context of HIV-1, some LRAs have the potential to induce DNAM-1 ligands on CD4^+^ T cells [200]; therefore, it will be important to evaluate the contribution of DNAM-1-mediated responses of NK cells against HIV-1 infection upon the “shock-and-kill” triggering. 

In the context of tumors, several chemotherapeutics compounds are known to be able to up-modulate on tumor cells, the expression of ligands for NK cell-activating receptors [203]. Viral infection often occurs in several immune-compromised tumor patients before and after chemotherapy or HSCT. Of note, several tumor malignancies are associated with viral infections. Then, the anticancer drug-mediated immunomodulating effects on NK cells in response to viral infections should be further evaluated [204].

Another anti-viral clinical intervention that merits consideration is the adoptive transfer of ex vivo activated and expanded NK cells, an immunotherapeutic approach that is acquiring great interest for the cure of hematological and solid tumors [205]. In the context of viral infection, it represents a promising clinical perspective aimed to promote the viral clearance for the cure of immune compromised patients [206] as well as chronically virus-infected patients [207]. Moreover, although it needs still further investigations, the use of chimeric antigen receptor (CAR)-engineered NK cells specifically committed to recognized and kill cells expressing viral proteins represents a novel, fascinating approach [208].
